# Dietary intake and blood lipid profile in overweight and obese schoolchildren

**DOI:** 10.1186/1756-0500-5-598

**Published:** 2012-10-30

**Authors:** Ana Elisa Madalena Rinaldi, Erick Prado de Oliveira, Fernando Moreto, Gleice Fernanda Costa Pinto Gabriel, José Eduardo Corrente, Roberto Carlos Burini

**Affiliations:** 1 CeMENutri – Centre for Physical and Nutritional Metabolism, Sao Paulo State University/UNESP, Department Of Public Health, Botucatu City, Sao Paulo State, Brazil; 2 School of Medicine, Federal University of Uberlândia, Uberlândia City, Minas Gerais State, Brazil; 3 Department Of Pathology, Sao Paulo State University/UNESP, Botucatu City, Sao Paulo State, Brazil; 4 School of Medicine, Southern Paraná State University (Unioeste), Cascavel city, Paraná State, Brazil; 5 Department Of Statistics, Sao Paulo State University/UNESP, Botucatu City, Sao Paulo State, Brazil

**Keywords:** Body mass index, Schoolchildren, Food consumption, Blood lipid

## Abstract

**Background:**

The high blood lipid levels and obesity are one of the main risk factors for cardiovascular diseases, and the atherosclerotic process begins in childhood. Some environmental factors are supposed to be involved in this relationship, such as dietary factors. This study aimed to investigate the relationship between dietary intake and blood lipids levels in overweight and obese schoolchildren.

**Methods:**

This is a cross-sectional study with 147 overweight and obese schoolchildren in Botucatu city, Brazil. The anthropometric measurements (body weight, height, body mass index, waist circumference and skinfolds), pubertal staging evaluation and biochemical tests were taken in all children. Three 24h-recall were applied in order to estimate the dietary intake and its relationship with blood lipid levels. The Student t test and multiple linear regression analysis were used for statistical analysis. Statistical significance was assessed at the level of 0.05. The data were processed in SAS software (version 9.1.3; SAS Institute).

**Results:**

At this study, 63% of children were obese (body mass index higher than 95^th^ percentile) and 80% showed high body fat percentage. The percentage of children with abnormal total cholesterol and triglycerides was 12% and 10%, respectively, and 28% presented at least one abnormal lipid levels. The average values of anthropometric measurements were higher in children with elevated lipid levels. Total cholesterol levels were positively related to full-fat dairy products and triglycerides levels to saturated fat percentage.

**Conclusions:**

Saturated fat was positively associated with elevated lipid levels in overweight and obese schoolchildren. These results reinforce the importance of healthy dietary habits since childhood in order to reduce the risks of cardiovascular diseases in adulthood.

## Background

Cardiovascular diseases (CVD) remain the number one cause of death in the United States [1] and in the industrialized Western World [2]. It is well established that atherosclerotic process begins in childhood and its severity is related to the presence and intensity of some risk factors, such as overweight/obesity, blood lipid levels, tobacco use, family history, blood pressure, dietary composition [3].

The prevention of cardiovascular diseases must begin decades prior to the onset of symptoms to be effective [4]. There are two types of prevention. The first one is prevent the development of risk factors and the second is manage those children with these risk factors who are identified before [3]. Optimal detection of dyslipidemia in youth includes both selective screening of those whose parent has premature CVD or dyslipidemia or who themselves have obesity, multiple CVD risk factors, diabetes, nephritic syndrome, and universal screening at the age of ten years [1].

Due to the scarcity of dyslipidemia longitudinal studies in childhood and adulthood cardiovascular diseases, there are several speculations [5]. Barker et al. (2007) [6] detected linear association between body mass index in aged 7 and 13 year and the ratio of heart failures. Furthermore, there is also an epidemiologic cardiovascular risk in children with dyslipidemia and tracking indicates that children maintain their percentile ranking over the time [7]. Among the risk factors tracking young age through adulthood, the obesity and cholesterol levels are the strongest ones [3].

The environmental factors, such physical activity and dietary factors, can influence the lipids levels and this relationship is known in adults [8,9]. However, there are few studies that evaluated the influence of dietary factors in childhood [10,11].

This study aimed to investigate the relationship of dietary intake and blood lipids levels in overweight and obese schoolchildren.

## Methods

### Study design and subjects

This is a cross-sectional study developed from June 2007 to August 2008 in three primary schools from Botucatu city, Sao Paulo State, Brazil. These schools were chosen based on previous approval of their principals and from the Municipal Education Department. The inclusion criteria was children with high body mass index (BMI) (BMI ≥85^th^ percentile), according to age and gender [12], that should undergo through biochemical tests. The exclusion criteria were overweight and obese children with any endocrine, renal, heart, or liver diseases and those that were undergoing through clinical nutritional care.

The study was developed in 4 phases: 1^st^) Anthropometric assessment in all schoolchildren enrolled in one of the schools (n=702). 2^nd^) The overweight and obese children (body mass index ≥85^th^ percentile) were contacted by telephone in order to participate of the next phases of the study (dietary intake assessment, clinical examination and biochemical tests) (n=246). After the contact, one child was eliminated because of the exclusion criteria (hypothyroidism), 62 children did not agree on participate in the next phase and six children dropped out the schools (n=177). 3^rd^) At this phase, children underwent through biochemical tests and answered the second 24-hour recall (n=177). A total of 22 children were excluded because did not collect blood samples (n=155). 4^th^) Finally, children answered the third 24-hour recall (n=155). Seven children were excluded because did not complete the three 24-hour recalls (n=147). One hundred and forty-seven overweight children, from 6 to 10 years old, participated of all study phases.

This study was approved by the Research Ethics Committee of Botucatu School of Medicine, State University of Sao Paulo, according to official letter number 579/2006 (protocol number). The parents or legal guardians signed a written informed consent.

### Anthropometric measurements and pubertal-stage assessment

The measurements were performed by trained professionals. All anthropometric measures were taken twice and the average number was considered. Body weight was measured using electronic scale (Filizola®) to the nearest 0.1 kg while the schoolchildren were barefoot and wearing light clothes. Height was measured by using a portable stadiometer (Seca®) to the nearest 0.1cm. Body mass index (BMI) was calculated by using weight and height data converted into percentiles according to age and gender [12] using EpiInfo software (version 3.3) [13]. Children were classified as overweight and obese according to BMI (overweight: BMI ≥85th percentile and <95th percentile; obese: BMI ≥95th percentile). Waist circumference (WC) was measured on the midway between rib cage and superior border of the iliac crest after complete expiration by using a measuring tape (Sanny®). The triceps (TSF) and subscapular (SSSF) skinfolds thickness were measured to the nearest 1.0 mm on the right side of the body by using a caliper (Lange®), according to World Health Organization [14]. Body fat percentage was estimated from skinfolds thickness measurements using the equations proposed by Slaughter et al. (1988) [15] and the reference values proposed by Lohman (1987) [16] (high body fat percentage: ≥25% for girls and ≥ 20% for boys). Pubertal stage was assessed by a pediatrician using a 5-stages scale for breast development and pubic hair in girls; testicular size and pubic hair in boys, according to the method of Tanner (1962). The children were thereafter classified in pre pubertal (stage 1) and pubertal (stages 2–4) [17].

### Biochemical tests

The children were submitted to vacuum venous puncture after 12-hour overnight fast. Triglycerides, total cholesterol and high density lipoprotein-Cholesterol (HDL-C) were assayed in the same day. Cholesterol and triglycerides levels were measured by enzymatic procedures in a semi-automatic spectrophotometer (Labquest, Labtest Diagnostica). Low density lipoprotein-Cholesterol (LDL-C) was calculated by difference from assayed total and HDL-Cholesterol and tryglicerides in the Friedewald formula [18]. According to the American Heart Association, the cutoff points for elevated lipids levels were total cholesterol ≥5.17mmol/L, LDL cholesterol ≥3.36mmol/L, HDL <0.91 mmol/L and triglycerides ≥1.70mmol/L [19].

### Dietary intake assessment

Three non-consecutive 24-hour recalls were applied, two referring to weekdays and one to the weekend, whereby all the foods and drinks consumed on the previous day were recorded to the interviewer. The 24-hour recalls were applied in the presence of child’s parents or legal guardians because they can report dietary intake with more reliability than the children [20]. Recent systematic review study concluded that 24-hour recall cover at least three days (weekday and weekend days) with parents as reporters is the best method to evaluated energy intake in children aged 4 to 11 years [21]. The information was recorded in household measures servings and thereafter it converted into grams and milliliters [22-24]. It was used a photographic manual of food portion size to improve the amount of dietary intake [25]. Dietary intake data were analysed using NutWin 1.5 software [15], a Brazilian software that included specific Brazilian foods and United State Department of Agriculture (USDA) nutrient database. The dietary intake was evaluated using Acceptable Macronutrient Distribution Range (AMDR-DRIs, 2002), that is a range of intake for a macronutrients, expressed as a percentage of total energy intake (%TEI) and that is associated with reduced risk of chronic disease while providing adequate intakes of essential nutrients. The dietary cholesterol, percentage of fats (saturated, monounsaturated and polyunsaturated) and sugar from TEI was evaluated according to World Health Organization (WHO, 2003) and fiber according to Williams (1992). Percentage of sugar from TEI included all food that the main ingredient is sucrose.

All the foods recorded in 24h-recalls were classified in eight food groups from the Brazilian food pyramid [16], which were: cereals (rice, pasta, oatbran, breads, wheat flour, corn); fruits; vegetables; legumes (any kind of beans, chicken pea, lentils); full-fat dairy products (cheese, whole milk, whole yogurt), meat (red meat, chicken, eggs, fish, processed meat, sausage), oils and fats (vegetable oil, olive oil, margarine, butter) and sugars and sweet foods (sucrose, honey, sodas, sweetened beverages, candies and lollypops, chocolate and chocolate drinks). These food groups are based in MyPyramid food guidance [26].

### Statistical analysis

The normality of data was evaluated by the Shapiro-Wilk test. The anthropometric measurements and blood lipids were expressed as means and standard deviation and dietary intake (energy, macronutrients, cholesterol, fiber and food groups) were described in median and interquartile range (first and third quartile). The Student t test was used to compare anthropometric measurements and lipid levels by gender and to compare anthropometric measurements by categorized blood lipids levels (elevated *vs* normal levels). The analysis of variance (ANOVA) with Tukey test was used to compare lipid levels by pubertal stage with adjust for age. The Tukey test was selected because it is possible to adjust for covariates.

The probability of adequate intake of energy, macronutrients, fibers and cholesterol was evaluated after intraindividual variability adjustment using Software for Intake Distribution Estimation (PC-SIDE version 1.02, 2003; Department of Statistics, Iowa State University).

To evaluate the relationship between dietary intake (food groups and total energy intake percentages of macronutrients) and total cholesterol, triglycerides, LDL-Cholesterol and HDL-Cholesterol (in a categorized model – elevated and normal levels) multiple linear regression analysis was used adjusted for the sex, gender, TEI and BMI. The estimation of foods that are eaten sporadically is more complex in statistical modeling due to the higher frequency of consumption equal to zero (Tooze et al., 2006) [27]. In this study, the consumption of some food groups (fruits and vegetables) was sporadic (zero or near zero). For this type of analysis was used is a statistical method developed by the National Cancer Institute (NCI) that estimates the distribution of food intake sporadically consumed from data of two or more 24h-recalls. This statistical model represents the habitual food consumption as a result of the probability of the food intake amount in a given day. The food amount intake was transformed to normal distribution using the Box-Cox transformation. A multiple linear regression analysis was performed by a routine commands (MIXTRAN DISTRIB) available on NCI website to identify the habitual food intake and its association with covariates. The same analysis was performed to evaluate the relationship between dietary intake and blood lipid levels.

The accepted level of significance was of 0.05. The data were processed in SAS software (version 9.1.3;SAS Institute).

## Results

One hundred and forty-seven overweight and obese schoolchildren participated in the study, presenting homogenous distribution for gender (52% girls). Among all schoolchildren that agreed with participating to the study, 55 were classified as overweight (BMI between the 85^th^ and 95^th^ percentile) and 92 were obese (BMI≥95^th^ percentile). Most of the children (80%) showed high body fat percentage and the mean values were similar for both genders. As regards pubertal stage, 98 (67%) children were classified as pre-pubertal (stage 1) and 49 (33%) as pubertal (stages 2, 3 and 4). The pubertal stage was described in two categories because only two and four children were classified in Tanner’s stage 3 and stage 4, respectively. No child was classified as post-pubertal (stage 5).

No differences were observed for age, anthropometric and biochemical data between girls and boys. However, pubertal children had higher body weight, height and adiposity indicators (BMI, WC and %BF) and low HDL-C levels than pre-pubertal children even after adjustment for age (Table 1). The adjustment by age was applied because pubertal maturation was correlated with age, so the main focus was evaluated the isolated relationship between pubertal stage and blood lipid levels.

**Table 1:** Anthropometric measurements and lipid levels according to gender and puberty stage in overweight schoolchildren

**Variables**	**Total (n=147)**	**Boys (n=71)**	**Girls (n=76)**	**p-value**	Pre-pubertal (n=98)**	**Pubertal (n=49)**	**p-value**
	**Mean(SD)**	**Mean(SD)**			**Mean(SD)**		
Age (years)	7.9(1.4)	7.9(1.5)	7.9(1.4)	0.88	7.5(1.3)	8.9(1.0)	0.000
Body weight (kg)	40.3(10.7)	41.6(11.6)	39.1(9.7)	0.14	37.4(10.4)	46.2(8.8)	0.000
Height (cm)	134.1(10.4)	135.3(10.6)	132.9(10.2)	0.18	130.6(9.6)	141.1(8.2)	0.000
WC (cm)	71.3(9.2)	72.8(10.2)	70.0(8.0)	0.07	69.6(9.3)	74.9(7.9)	0.000
Body fat (%)	28.7(6.2)	29.1(7.0)	28.3(5.5)	0.43	27.8(6.8)	30.5(4.5)	0.005
TC (mmol/L)	4.28(0.06)	4.26(0.08)	4.29(0.09)	0.81	4.29(0.07)	4.23(0.11)	0.654
HDL-C (mmol/L)	1.26(0.02)	1.26(0.03)	1.25(0.04)	0.76	1.29(0.03)	1.19(0.03)	0.044
LDL-C (mmol/L)	2.55(0.05)	2.52(0.07)	2.58(0.07)	0.58	2.55(0.05)	2.54(0.1)	0.791
TG (mmol/L)	1.03(0.05)	1.04(0.07)	1.03(0.06)	0.94	0.98(0.05)	1.12(0.21)	0.177
	**Number and percentage of schoolchildren n(%)**						
BMI(≥85^th^ and <95^th^ percentile)^1^	55 (37.4)	21(29.6)	34(44.7)	---	37(37.8)	18(36.7)	---
BMI (≥95^th^ percentile)^1^	92(62.6)	50(70.4)	42(55.3)	---	61(62.2)	31(63.3)	---

*BMI*: body mass index; *HDL-C*: high density lipoprotein cholesterol; *LDL-C*: low density lipoprotein cholesterol; *SD*: standard deviation; *TC*: total cholesterol; *TG*: triglycerides; *WC*: waist circumference.

**The comparison between pre-pubertal and pubertal children was ajusted by age (Tukey Test).

1: percentages of children according BMI classification by gender and puberty stage (only descriptive results).

The elevated total cholesterol and triglycerides levels were the most frequent in plasma, followed by the others. The frequency of children that presented at least one of the elevated lipid levels was 28%, with just one elevated lipid level was 17%, with two elevated lipid levels was 10% and only one child presented elevated lipid levels (TG, total cholesterol and HDL-C). Children with elevated triglycerides levels had higher average weight, WC and %BF, but no difference was observed for total cholesterol, LDL-C and HDL-C (Table 2).

**Table 2:** **Anthropometric measurements according to lipid levels (elevated *vs *normal levels) in overweight schoolchildren**

**Variables**	**Total Cholesterol (mg/dL)**		**LDL-Col (mg/dL)**		**HDL-Col (mg/dL)**		**Triglycerides (mg/dL)**	
	**Normal(n=130)**	**Elevated(n=17)**	**Normal(n=134)**	**Elevated(n=13)**	**Normal(n=134)**	**Elevated(n=13)**	**Normal(n=132)**	**Elevated(n=15)**
	**Mean(SD)**		**Mean(SD)**		**Mean(SD)**		**Mean(SD)**	
**Weight (kg)**	40.0(10.7)	42.4(11.2)	40.0(10.6)	43.0(12.1)	40.0(10.8)	43.2(9.3)	39.4(10.4)	47.9(10.4)
p-valor	0.3929		0.3425		0.3078		**0.0032**	
**WC (cm)**	71.2 (9.1)	72.7(10.0)	71.1(9.1)	73.6(10.2)	71.1(9.3)	73.5(7.9)	70.5(8.9)	78.6(8.8)
p-valor	0.5112		0.3548		0.3919		**0.0012**	
**Body Fat (%)**	28.5 (6.3)	30.2(6.0)	28.6(6.3)	29.8(5.6)	28.6(6.3)	29.3(5.7)	28.1(6.0)	34.0(6.5)
p-valor	0.2917		0.4896		0.7268		**0.0004**	

*HDL-C*: high density lipoprotein cholesterol; *LDL-C*: low density lipoprotein cholesterol*; WC:* waist circumference.

The dietary intake was described in Table 3 and was similar by gender (data not shown). The dietary intake of saturated fat, percentage of sugar, oils and fats (servings) and sugars/sweet foods (serving) was higher than the recommendations. The intake of fruits, vegetables and cereals was very low (Table 3). The full-fat dairy products were lower than recommendations, but higher than other food groups. The probability of adequacy was high (higher than 95%) for carbohydrate, protein, total fats and cholesterol, but low for polyunsaturated fat (50%), monounsaturated fat (6%) and saturated fat (37%). The probability of adequacy for fiber was evaluated according age and the probability of adequacy reduced according the increase of age (53% for aged 6 and 5% for aged 10) (data not shown).

**Table 3:** Dietary intake characteristics across the 24h-recall in overweight schoolchildren

Dietary variables*	**Median (1st-3rd quartile) (n=147)**	**Recommendation**
Total energy intake (kcal)	1591 (1410–1884)	------
Carbohydrate (%TEI)	53(49–57)	45-65%*
Sugar (%TEI)	13(9–17)	<10%**
Protein (%TEI)	16(14–18)	10-30%*
Total Fat (%)	31(28–34)	25-35%*
Saturated fat (%TEI)	12(10–13)	7-10%**
Monounsaturated fat (%TEI)	8(7–10)	10-20%**
Polyunsaturated fat (%TEI)	6(5–7)	6-10%**
Cholesterol (miligrams)	193(158–250)	<300mg**
Fiber (grams)	11(8–13)	5g+age***
Cereals (servings)	3(2–4)	6 servings****
Fruits (servings)	0.5(0–1)	4 servings****
Vegetables (servings)	0.5(0.2-0.9)	4 servings****
Legumes (servings)	0.6(0.2-1.0)	1 serving****
Full-fat dairy products (servings)	1.4(0.7-2.1)	3 servings****
Meats (servings)	1.4(1.0-1.8)	2 servings****
Oils and fats (servings)	1.6(1.0-2.0)	1 serving****
Sugars and sweet foods (servings)	1.9(1.1-2.7)	2 servings****

%TEI: percentage of total energy intake.

*Acceptable macronutrients distribution range (AMDR-DRI, 2002); **World Health Organization (WHO, 2003); ***Williams (1992); ****Brazilian food pyramid (Philippi et al., 1999).

There was a positive relationship between elevated total cholesterol levels and full-fat dairy products and elevated triglycerides levels and saturated fat percentage of total energy intake (Table 4).

**Table 4:** Multivariate linear regression of dietary intake and blood lipid levels in overweight schoolchildren

**Dietary intake**	**Total Cholesterol**		**LDL-C**		**HDL-C**		**Triglyderides**	
	*β *(CI)*	**p**	*β *(CI)*	**p**	*β *(CI)*	**p**	*β *(CI)*	**p**
CHO (%)	−1.40(−4.85;2.32)	0.43	−0.89(−4.38;2.60)	0.62	−0.88(−4.31;2.55)	0.61	−0.72(−4.03;2.59)	0.67
PTN (%)	0.08(−0.19;2.18)	0.56	0.20(−0.13;0.53)	0.26	0.12(−0.17;0.41)	0.43	−0.01(−0.30;0.28)	0.95
Total Fat (%)	0.08(−0.06;2.11)	0.20	−0.03(−0.19;0.13)	0.70	0.02(−0.12;0.17)	0.83	0.03(−0.12;0.16)	0.75
SF (%TCI)	0.08(−0.08;2.12)	0.35	−0.05(−0.23;0.13)	0.62	0.03(−0.15;0.21)	0.75	0.17(−0.01;0.35)	0.04
MF (%)	0.13(0.03;2.17)	0.12	0.06(−0.12;0.24)	0.52	0.11(−0.07;0.29)	0.22	0.01(−0.17;0.19)	0.93
PF (%)	0.07(−0.20;2.17)	0.59	0.18(−0.13;0.49)	0.25	−0.00(−0.29;0.29)	0.98	−0.01(−0.30;0.28)	0.97
Cholesterol (mg)	−0.52(−2.42;2.41)	0.59	0.43(−1.75;2.61)	0.69	−1.51(−3.65;0.63)	0.17	1.47(0.65;3.59)	0.18
Fiber (g)	−0.01(−0.50;2.20)	0.98	0.08(−0.47;0.63)	0.78	0.11(−0.42;0.64)	0.70	−0.16(−0.73;0.33)	0.56
Cereal (servings)	−0.06(−0.41;2.08)	0.77	0.08(−0.33;0.49)	0.69	−0.05(−0.44;0.34)	0.81	−0.06(−0.45;0.33)	0.75
Meat (servings)	0.03(−0.21;2.11)	0.80	0.19(−0.08;0.46)	0.17	0.08(−0.18;0.34)	0.57	0.06(−0.20;0.32)	0.66
Legumes(servings)	0.10(−0.29;2.26)	0.63	0.20(−0.21;0.61)	0.33	−0.08(−0.47;0.31)	0.69	−0.26(−0.65;0.13)	0.21
Vegetables (servings)	−0.21(−0.80;2.05)	0.49	0.26(−0.33;0.85)	0.39	0.22(−0.45;0.89)	0.52	0.07(−0.58;0.72)	0.84
Fruits (servings)	−0.12(−0.65;2.11)	0.65	−0.24(−0.85;0.37)	0.43	−0.37(−0.98;0.24)	0.24	−0.23(−0.84;0.38)	0.45
Full-fat dairy products (servings)	0.36(0.01;2.50)	0.04	−0.38(−0.77;0.01)	0.06	−0.03(−0.42;0.36)	0.88	0.18(−0.19;0.55)	0.35
Sugars and sweet foods (servings)	−0.04(−0.61;2.21)	0.90	−0.09(−0.74;0.56)	0.77	−0.04(−0.67;0.59)	0.90	0.18(−043;0.79)	0.56
Oils and fats (servings)	0.33(−0.73;2.83)	0.54	0.57(−0.65;1.79)	0.36	0.40(−0.74;1.54)	0.50	−0.08(−1.20;1.04)	0.89

*CHO*: carbohydrate; g: grams; *HDL-C: HDL* cholesterol; *LDL-C: LDL* cholesterol; *MF*: monounsaturated fat; *PF*: polyunsaturated fat; *PTN*: protein; *SF*: saturated fat.

*TC*: total cholesterol; *TG*: triglycerides.

*Adjusted by: age, gender, body mass index, total energy intake.

## Discussion

The high total cholesterol and high triglycerides levels were the two main alterations in blood lipids and there are associated with dietary saturated fat. The present study investigated only overweight and obese children and found that average of body weight, waist circumference and body fat percentage were higher in children with elevated triglycerides level. The inclusion criteria of children in this study agreed to the recent American Academy of Pediatric recommendation [10] (fasting lipid profile screening for youths with BMI≥85^th^ percentile on the CDC growth charts [17]).

In Brazil, there is not a national study that describes the blood lipid profile in youths. The compilation of the most recent Brazilian regional studies showed that the prevalence of high lipid and lipoprotein levels ranges from 3.1 to 43.8% in children and adolescents [28-31]. However, it is difficult to compare these results among the studies because of the different reference values and cutoff point adopted - Brazilian Heart Society, American Academic of Pediatrics, National Cholesterol Education Program.

The prevalence of high total cholesterol (10%) and triglycerides (9.7%) in United States pediatric population was similar to the current study as well as the reference and cutoff point adopted. These authors showed that high adiposity was associated with high blood lipid levels and the body fat percentage can explain until 20% of the plasma lipid levels, although the authors explain that there is no clear definition of too much body fat percentage in children [44]. In the current study, children with high triglycerides levels had higher body weight, WC and body fat percentage. This result can be explained because adiposity is strongly related to higher triglycerides levels in children and adults. This relationship is weaker between adiposity and LDL-Cholesterol [27]. I’Allemand et al. (2008) [32] showed higher percentage of high HDL-Cholesterol and triglycerides levels in obese than eutrophic children, but no difference for LDL-Col and total cholesterol. Beyond evaluation overweight/obese through BMI, the body fat percentage is also related to blood lipid profile.

Dietary patterns are also a major contributing factor to the development of CVD [33] in association with other risk factors. The dietary fats are the most investigated and fully defined factors [34,35]. In this study, plasma triglyceride and total cholesterol were associated with dietary intake, specifically with full-fat dairy products and saturated fat. The dietary intake of saturated fat was higher than the recommendation (only 37% of children showed an adequate consumption) and dietary intake of monounsaturated and polyunsaturated fats were lower than the recommendation for almost all children. Although the serving number of full-fat dairy products was lower than adequate recommendation, this food group was related to high cholesterol levels. Alcantara-Neto et al. (2012) [31] showed similar association between high total cholesterol and high triglycerides with dietary saturated fat. Nicklas et al. (2002) and Sanchez-Bayle et al. (2008) [36,37] also showed a positive relationship between total cholesterol and dietary saturated fat. Other study showed lower concentration of total cholesterol in children that had consumed lower amount of saturated fat [38]. The saturated fat intake limited to 7 to 10% of TEI showed positive results in lower total cholesterol and LDL-C [3] in children. One of the good strategies to improve the blood lipid profile is changing full-fat dairy products to skim dairy products [39].

Full-fat dairy is one of the main sources of saturated fat, that increases the visceral adipose tissue in greater proportion than other fat types, since it reduces the activation of PGC-1α (PPARγ-coactivator), an important oxidative metabolism regulator. Thus, fatty acid oxidation is decreased, and accumulation in tissues and circulation is increased [40]. Hence, saturated fat intake would increase the visceral adipose tissue, which contributes to dyslipidemia. However, our results showed that the saturated fat is positively associated with high triglycerides concentration even when adjust for BMI, which shows that this type of fat can influence dyslipidemia by other mechanisms, without the influence of adiposity.

The current study showed some limitations that are explained below. This is a cross-sectional study and it is not recommended to establish a direct influence of the dietary composition on blood lipid profile. Another limitation was the lack of a control group comprising eutrophic children, which might have been possible to evaluate whether quality and amount of dietary intake would interfere in body weight/body adiposity or in blood lipid profile [41,42]. However, the recent recommendation is to screen overweight children in order to evaluate blood lipid profile [10].

As regards the diet, it was necessary to use nutritional information from labels for the industrialized foods. These labels in Brazil did not provide information concerning monounsaturated or polyunsatured fat; therefore, their intake may have been underestimated, particularly for the children who consumed large amounts of industrualized foods (high percentage of saturated fat). A recent Brazilian study [43] showed nutritional information inadequacy on the labels of snacks (saturated fat, fiber and sodium) and cookies (saturated fats); these foods were largely consumed by the children that participated of this study. Another possible limitation related to dietary intake was the underreporting, that is more frequent in overweight children compared to eutrophic ones [21].

## Conclusion

Full-fat dairy products and saturated fat were positively associated with total cholesterol and TG, respectively. These results indicate the importance of quality of food intake since childhood to reduce the risks of cardiovascular diseases in adulthood.

## Abbreviations

%BF: Body fat percentage; %TEI: Percentage total energy intake; BMI: Body mass index; HDL-C: High density lipoprotein-cholesterol; LDL-C: Low density lipoprotein-cholesterol; TEI: Total energy intake; WC: Waist circumference.

## Competing interests

The authors declare that they have no competing interests.

## Authors’ contributions

AEMR: conceived of the study, wrote the manuscript, acquired and organized the data and interpreted the results; EPO: wrote the manuscript and reviewed it. FM: carried out the biochemical measurements; GFCPG: carried out the anthropometric data and pubertal-stage assessment; JEC: performed the statistical analysis and interpreted the results. RCB: reviewed ion and approved the final version of the manuscript. All authors read and approved the final manuscript.
